# MAP17, a ROS-dependent oncogene

**DOI:** 10.3389/fonc.2012.00112

**Published:** 2012-09-06

**Authors:** Amancio Carnero

**Affiliations:** Instituto de Biomedicina de Sevilla, Hospital Universitario Virgen del Rocío, Consejo Superior de Investigaciones Cientificas, Universidad de SevillaSevilla, Spain

**Keywords:** MAP17, cancer, oncogene, reactive oxygen species, tumorigenesis

## Abstract

MAP17 is a small 17 kDa non-glycosylated membrane protein previously identified as being overexpressed in carcinomas. Breast tumor cells that overexpress MAP17 show an increased tumoral phenotype with enhanced proliferative capabilities both in the presence or the absence of contact inhibition, decreased apoptotic sensitivity, and increased migration. MAP17-expressing clones also grow better in nude mice. The increased malignant cell behavior induced by MAP17 is associated with an increase in reactive oxygen species (ROS) production, and the treatment of MAP17-expressing cells with antioxidants results in a reduction in the tumorigenic properties of these cells. The MAP17-dependent increase in ROS and tumorigenesis relies on its PDZ-binding domain because disruption of this sequence by point mutations abolishes the ability of MAP17 to enhance ROS production and tumorigenesis. MAP17 is overexpressed in a great variety of human carcinomas, including breast tumors. Immunohistochemical analysis of MAP17 during cancer progression demonstrates that overexpression of the protein strongly correlates with tumoral progression. Generalized MAP17 overexpression in human carcinomas indicates that MAP17 can be a good marker for tumorigenesis and, especially, for malignant progression.

The complex physiology of vertebrates requires the continuous renewal of most tissues, which may become damaged either by external agents or by the toxic byproducts of their own metabolism, to maintain homeostasis. Cancer arises as a consequence of genetic changes that deregulate the mechanisms that control the renewal process, either by activation of the pathways that promote survival and proliferation, or through inactivation of growth suppression pathways. In order for cancer cells to grow and metastasize, they must overcome additional barriers to their expansion by promoting angiogenesis, acquiring characteristics that allow them to survive in organs different from their origin or by evading immune surveillance mechanisms (Hanahan and Weinberg, 2000, 2011).

Tumorigenesis occurs when the mechanisms involved in the control of tissue homeostasis are disrupted and cells stop responding to physiological signals. Therefore, genes capable of desensitizing tumoral cells to physiological signals may provide a selective advantage within the tumoral mass and influence the outcome of the disease. We undertook a large-scale genetic screen to identify genes capable of altering the cellular response to physiological signals that resulted in a selective advantage during tumorigenesis (Hannon et al., 1999; Carnero et al., 2000; Vergel and Carnero, 2010). A genome-wide retroviral cDNA screen to search for genes that confer a selective advantage to cancer cells during tumorigenesis allowed us to identify MAP17 (Guijarro et al., 2007a). MAP17 is a small non-glycosylated membrane-associated 17 kDa protein that localizes to the plasma membrane and the Golgi apparatus (Blasco et al., 2003). The MAP17 protein sequence contains two transmembrane regions and a hydrophobic amino-terminus encoding a PDZ-binding domain (Jaeger et al., 2000; **Figure 1**). MAP17 overexpression in carcinomas was first described by using the technique of differential display (Kocher et al., 1995). MAP17 binds several PDZ domain-containing proteins, including NHeRF proteins, NaPi-IIa, and NHe3. Overexpression of MAP17 in opossum kidney cells participates in NaPi-IIa internalization to the *trans*-Golgi network (Lanaspa et al., 2007). In a transgenic mouse model, MAP17 hepatic overexpression resulted in PDZK1 (NHeRF3) liver deficiency, suggesting that MAP17 is an endogenous regulator of PDZK1 turnover (Silver et al., 2003). MAP17 acts as an atypical anchoring site for PDZK1 and interacts with the NaPi-IIa/PDZK1 protein complex in renal proximal tubular cells (Pribanic et al., 2003). The physiological role of MAP17 in proximal tubules is not known, but it does stimulate specific Na-dependent transport of mannose and glucose in *Xenopus* oocytes (Blasco et al., 2003) and some human cells (Guijarro et al., 2007a). The MAP17 gene does share regulatory elements with the stem cell leukemic gene (SCL, TAL-1), which encodes a basic Helix-Loop-Helix protein essential in the formation of the hematopoietic lineages (Gottgens et al., 2002; Delabesse et al., 2005). However, both genes show independent regulation (Guijarro et al., 2007c).

**FIGURE 1:**
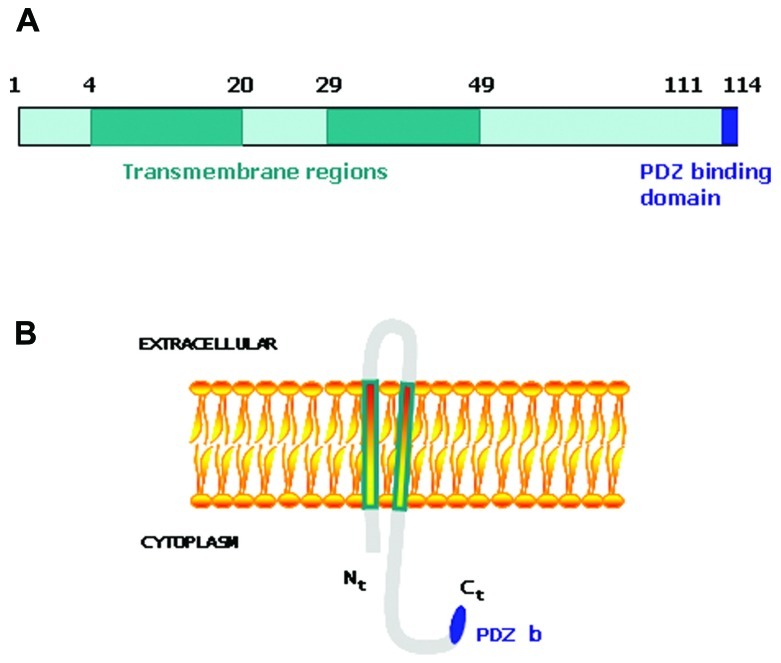
**(A)** Schematic representation of MAP17 protein domains. **(B)** Schematic representation of MAP17 disposition in the membrane.

Multiple oncogenes that activate signaling pathways directly involved in cell survival or proliferation have been discovered in previous decades. Other genes may provide an advantage to the tumoral cells, making them insensitive to physiological signals or altering their normal physiology. Although activated macrophages destroy cancer cells more effectively than normal cells, the ability to escape activated macrophages is a characteristic of tumor cells. One of the mechanisms responsible for the specific killing of tumor cells by macrophages is the production of the cytokine tumor necrosis factor-alpha (TNF-α). Therefore, resistance to TNF may provide cancer cells with a selective advantage against host elimination. Ectopic expression of MAP17 in tumor cells prevents TNF-induced G1 arrest by impairing p21waf1 induction. However, expression of MAP17 does not inhibit TNF-induced apoptosis in Me180-sensitive tumor cells. The inhibition of TNF is specific because MAP17 does not alter the response to other cytokines such as IFN-α. As described in the *Xenopus* oocyte system, MAP17 increases the uptake of glucose in some cells, but this effect is not responsible for TNF bypass.

## MAP17 IN HUMAN TUMORS

MAP17 overexpression in carcinomas occurs mostly through mRNA amplification, but promoter activation has also been observed by some oncogenes (Kocher et al., 1995; Guijarro et al., 2007c). Immunohistochemical analysis of MAP17 during cancer progression shows that overexpression of the protein strongly correlates with tumoral progression. Generalized MAP17 overexpression in human carcinomas indicates that MAP17 can be a good marker for tumorigenesis and especially for malignant progression.

**FIGURE 2:**
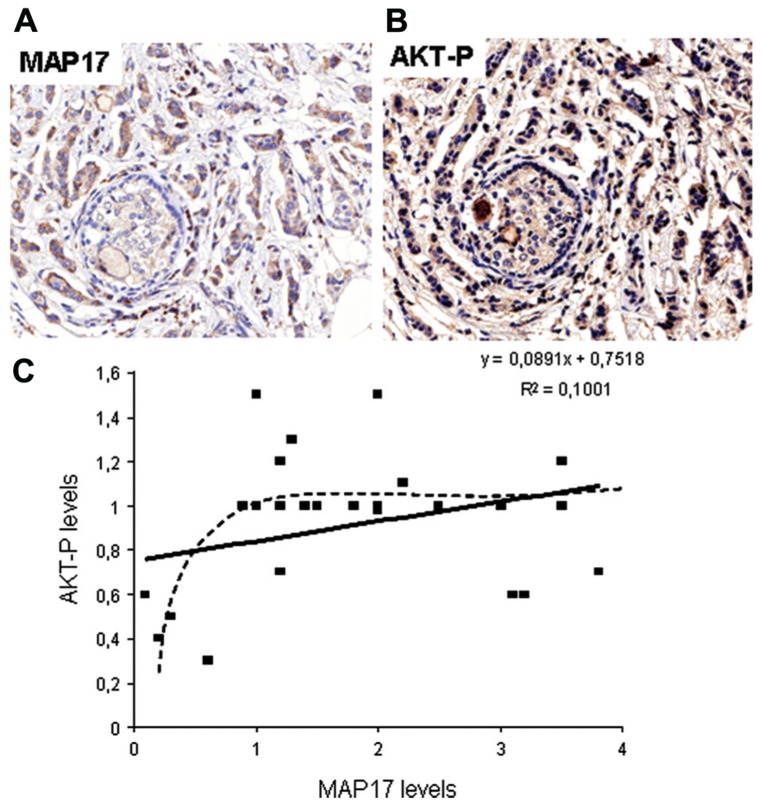
**(A)** Representative picture of human breast tumors overexpressing MAP17. **(B)** Same tumor sample showing activated AKT (phosphorylated at S473). **(C)** Correlation between MAP17 expression and AKT activation in breast tumor samples analyzed.

MAP17 is highly expressed in renal proximal tubular cells and has been previously described to be associated with carcinomas (Kocher et al., 1995, 1996). We have performed an in-depth analysis of MAP17 overexpression in carcinomas by immunohistochemistry and mRNA expression (**Figure 2**). We have found that the MAP17 protein is overexpressed in a large percentage of the tumors analyzed and is significantly correlated with the tumor grade in ovarian, breast, and prostate carcinomas (Guijarro et al., 2007c, 2012). The analysis of mRNA levels by Q-PCR or by hybridization comparing tumoral vs. non-tumoral tissues of the same patient, demonstrate an even higher percentage of tumor samples with MAP17 overexpression. In tumors such as ovary, colon, stomach, cervix, and thyroid gland, the percentage of overexpression in tumor samples is higher than 70%, while in lung, uterus, and rectum it is approximately 50%. Although more samples need to be analyzed to confirm these high rates, the data suggest that MAP17 overexpression is the most common marker of tumorigenesis in carcinomas. The relevance of MAP17 as a general marker for the malignant stages of human tumors still needs to be confirmed in additional tumor types and larger cohorts. However, all tissues explored thus far have shown similar patterns of MAP17 expression. Furthermore, MAP17 expression seems to correlate with AKT phosphorylation at Ser473 (**Figure 2**). These expression patterns provide a mechanistic insight and a possible target for future therapies (AKT inhibition).

## ONCOGENIC ACTIVITY OF MAP17

Tumor cells that overexpress MAP17 show an increased tumoral phenotype with enhanced proliferative capabilities both in the presence or absence of contact inhibition, decreased apoptotic sensitivity, and increased migration. MAP17-expressing clones also grow better in nude mice. The increased malignant cell behavior induced by MAP17 is associated with an increase in reactive oxygen species (ROS) production, and the treatment of MAP17-expressing cells with antioxidants results in a reduction in the tumorigenic properties of these cells. Treatment of breast cells with inhibitors of Na^+^-coupled co-transporters leads to an inhibition of a ROS increase and a decrease in the malignant cell behavior in MAP17-expressing clones (Guijarro et al., 2012). Finally, MAP17-dependent increase in ROS and tumorigenesis are dependent on its PDZ-binding domain because disruption of this sequence by point mutations abolishes the ability of MAP17 to enhance ROS production and tumorigenesis (Guijarro et al., 2007b). Furthermore, expression of a MAP17 specific shRNA in protein-expressing tumor cells reduced their tumorigenic capabilities (Guijarro et al., 2012), which suggests that this effect is dependent upon MAP17 protein expression.

**FIGURE 3:**
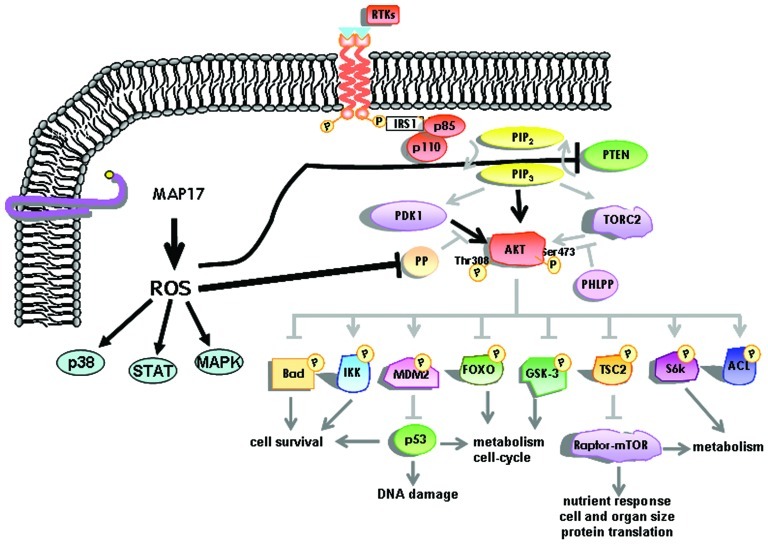
**Schematic representation of the intracellular pathways activated by MAP17 through ROS.** The AKT pathway is represented in more detail.

MAP17 significantly decreases the c-Myc induced caspase-3-like activity in Rat1 fibroblasts under low serum conditions. This decrease is in keeping with the concept of MAP17-induced PI3K/AKT signaling, in which MAP17 is able to interfere with Bax translocation to the mitochondria (Guijarro et al., 2007d). At the molecular level we have found that MAP17 protects Rat1a fibroblasts from Myc-induced apoptosis through, ROS-mediated activation of the PI3K/AKT signaling pathway (Guijarro et al., 2007d). A fraction of PTEN protein undergoes oxidation in MAP17-overexpressing cells. Furthermore, activation of AKT by MAP17 as measured by Thr308 phosphorylation was independent of PI3K activity (**Figure 3**). Importantly, modulation of ROS by antioxidant treatment prevented activation of AKT, thus, restoring the level of apoptosis in serum starved Rat1/c-Myc fibroblasts (Guijarro et al., 2007d). MAP17-mediated survival was associated with an absence of Bax translocation to the mitochondria and reduced caspase-3 activation. Finally, overexpression of a dominant negative mutant of AKT in MAP17-expressing clones makes them sensitive to serum depletion (Guijarro et al., 2007d). The data indicates that MAP17 protein activates AKT through ROS, and this activation is a determinant in conferring resistance to Myc-induced apoptosis in the absence of serum. These results might provide the mechanistic insight to explain the correlation between MAP17 levels and AKT phosphorylation found in tumor samples. Like ways, AKT activation has been described as responsible for TNF resistance in some tumor cell lines (Sudheerkumar et al., 2008; Xu et al., 2012).

## MAP17 IS A ROS-DEPENDENT ONCOGENE

The increased tumorigenic properties induced by MAP17 are associated with an increase in ROS because MAP17 increases endogenous ROS and the antioxidant treatment of MAP17-expressing cells entails a reduction in the tumorigenic properties of these cells. Two explanations can be offered for the mechanism by which ROS induce the transformed phenotype. First, reactive oxygen generated in the presence of MAP17 may be mutagenic, causing the transformed phenotype through the induction of mutations in oncogenes or tumor suppressor genes. Alternatively, ROS generated in a MAP17-dependent manner might function as an intracellular signal, inducing a growth-related genetic program. We have found that ROS removal by antioxidant treatments decrease the malignant cell behavior induced by MAP17; thus, the second hypothesis is favored. Accumulating evidence implicates ROS in signaling cascades related to cell proliferation and transformation (Sundaresan et al., 1995; Burdon, 1996; Irani et al., 1997). Ras-transformed fibroblasts overproduce ROS, and this overproduction is correlated with the activation of mitogenic signaling pathways (Irani et al., 1997). Loss of superoxide dismutase (SOD; which should elevate ROS levels) has also been correlated with a tumoral phenotype, and overexpression of SOD leads to the reversion of the transformed phenotype (Fernandez-Pol et al., 1982; Church et al., 1993; Yan et al., 1996). On the other hand, H_2_O_2_ is generated in response to the growth factors EGF and PDGF and is linked to growth-related signaling (Sundaresan et al., 1995; Bae et al., 1999). When overexpressed in NIH3T3 mouse fibroblasts, Nox1, a NADPH oxidase catalytic subunit, induces excessive production of ROS and a transformed phenotype with increased mitotic rates and aggressive tumor formation in athymic mice (Arnold et al., 2001). The phenotype of Nox1-transfected cells can be reversed by ROS reduction through stable expression of catalase, thereby implicating ROS as a signaling molecule (Arnold et al., 2001).

The cellular targets responsible for growth and transformation affected by ROS signaling are not fully known. DNA microarray experiments (Arnold et al., 2001) indicate that up to 2% of the genes are regulated by ROS. Furthermore, we have found that a ROS increase activates the PI3K pathway, which may be by direct oxidation and inactivation of PTEN and other AKT phosphatases, thus maintaining AKT activation even in the absence of a PI3K signal (Guijarro et al., 2007d). AKT pathway activation induced by MAP17 expression might explain some of the properties described here. However, we hypothesize that other pathways must coexist that are induced by MAP17 at the transcriptional level, as described in other systems (Klaunig et al., 1998; Droge, 2002). The p42/p44 mitogen-activated protein kinase (MAPK), p38 MAPK, p70S6k, AKT, and STAT, signaling pathways are all activated by ROS (Natarajan et al., 1993; Finkel, 1998; Bae et al., 1999; Allen and Tresini, 2000; Ray et al., 2012; Vurusaner et al., 2012). A variety of other targets can also be affected by ROS, including transcription factors such as NF-kB (Schmidt et al., 1995), AP1 (Wenk et al., 1999), and p53 (Hainaut and Milner, 1993). In most cases the activation is indirect (Min et al., 1998; Abe et al., 2000). However, a direct effect has been shown on protein tyrosine phosphatase-1B (PTP-1B), which is inhibited by oxidation of a thiol in the active site (Lee et al., 1998; Barrett et al., 1999), leading to increased phosphotyrosines on many cell proteins. ROS can directly modify signaling proteins through different modifications such as nitrosylation, carbonylation, disulfide bond formation, and glutathionylation (England and Cotter, 2005). Whatever the proximal target(s), ROS reprogram the expression of enzymes and other proteins in the cell (Klaunig et al., 1998; Droge, 2002).

However, the increased tumoral properties of carcinoma cells were not paralleled in immortal non-tumoral cells (Guijarro et al., 2012), indicating that MAP17 provides a selective advantage once tumorigenesis has begun. ROS act as a second messenger that enhances tumoral properties, but only in those cells where the senescence/apoptotic signal provided by ROS is uncoupled. In primary cells, MAP17 triggers a ROS-dependent, senescence-like response that is abolished in the absence of p38a activation. Furthermore, in human breast tumors, MAP17 activation is correlated with a lack of phosphorylation of p38a. Therefore, MAP17 is overexpressed in late-stage breast tumors, in which oncogenic activity relies on p38 insensitivity to induced intracellular ROS (Guijarro et al., 2012).

## MAP17 AND NHeRFs

MAP17 has been found to bind NHeRF1 and NHeRF3 (PDZK1) through its PDZ-binding motif (Pribanic et al., 2003; Silver et al., 2003; Lanaspa et al., 2007). NHeRFs are scaffolding protein defined by the presence of globular PDZ domains that assemble several proteins into functional complexes (Shenolikar et al., 2004; Cunningham et al., 2010; Claperon et al., 2011). The NHeRF proteins regulate cell surface expression and functional activity of transporters (Shenolikar et al., 2004; Lee et al., 2007). Most transporters identified as binding partners belong to the ABC family (Weinman et al., 2010). In addition to transporters, other proteins have been shown to interact with NHeRF proteins, including signaling proteins, hormone receptors, and cytoskeleton structural elements (Theisen et al., 2007). Many proteins related to the G-protein signaling pathways were found to interact with PDZK1, and they were likely to be functionally associated with transporters (Cardone et al., 2007; Theisen et al., 2007; Carnero, 2012). Furthermore, it has been shown (Dai et al., 2004) that NHeRF1 binds to the breast tumor suppressor SYK and MERLIN, the product of the tumor suppressor NF2. NHeRF1 present also mutations at the PDZ domains in breast tumors which abolishes binding to these suppressor proteins. Primary breast tumors with LoH at the NHeRF1 locus show higher aggressiveness. However, the relation of these mutations with MAP17 or other physiological alterations such as ROS of glucose uptake is at present unknown.

MAP17 form complexes with PDZK1 and NHe3 contributing to basal and calcium inhibition of NH3 activity (Cinar et al., 2007). Recently, it has been shown that PDZK1 regulates PLC β3 (Kim et al., 2012). PDZK1 also regulates the solute carriers PEPT1 (oligopeptide transporter) and OCTN2 (carnitine/organic cation transporter; Sugiura et al., 2008), the cystic fibrosis transmembrane conductance regulator (CFTR; Gentzsch et al., 2003), canalicular multispecific organic anion transport (CMOAT; Inoue et al., 2004), and the anion exchangers of the SLC26A family (Hillesheim et al., 2007). PDZK1 has also been shown to interact with AKAP10, FARP2, sodium–hydrogen antiporter 3 regulator 1, SLC22A12, SLK, SLC22A4, and SLC34A3 (Counillon et al., 2000; Gisler et al., 2003; Shenolikar et al., 2004; Ganapathy et al., 2008). As has been suggested, it is possible that the role of MAP17 is to enhance the endogenous uphill transport system (Blasco et al., 2003; Cardone et al., 2005; Chiche et al., 2010; Parks et al., 2011; Carnero, 2012).

**FIGURE 4:**
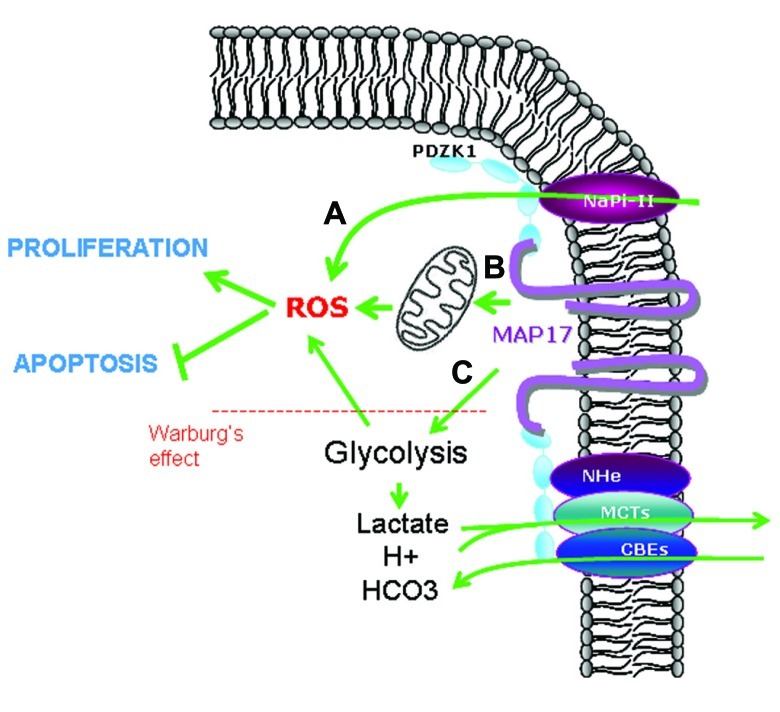
**Possible mechanisms involved in MAP17-dependent increase of ROS.** **(A)** Direct pH alteration by membrane transports, **(B)** increase in glucose metabolism through mitochondrial respiration, **(C)** increase in aerobic glycolysis (Warburg’s effect) which is allowed by acidic detoxification carried out by membrane transports bund to MAP17–NHeRFs complexes.

On the other hand, MAP17 has been shown also to increase glucose uptake (Blasco et al., 2003; Guijarro et al., 2007a) thus enhancing glycolysis, contributing to Warburg’s effect and increasing intracellular oxidative stress (Bar-Even et al., 2012; Carnero, 2012). Therefore, MAP17 increase in tumor cells could be a mechanistic advantage that will permit tumor cells increase the glucose intake and in parallel decrease the intracellular pH and lactic acid by the increase of membrane bound transports (Carnero, 2012; **Figure 4**).

## CONCLUDING REMARKS

In summary, MAP17 overexpression in human breast carcinomas indicates that MAP17 can be a good marker for tumorigenesis and for malignant progression. Our results indicate that this protein is likely to play an important role in carcinogenesis.

## Conflict of Interest Statement

The author declares that the research was conducted in the absence of any commercial or financial relationships that could be construed as a potential conflict of interest.
